# Anti-tumor efficacy of anti-GD2 CAR NK-92 cells in diffuse intrinsic pontine gliomas

**DOI:** 10.3389/fimmu.2023.1145706

**Published:** 2023-05-12

**Authors:** Pengcheng Zuo, Yaopeng Li, Chi He, Tantan Wang, Xu Zheng, Hao Liu, Zhen Wu, Junting Zhang, Xuebin Liao, Liwei Zhang

**Affiliations:** ^1^ Department of Neurosurgery, Beijing Tiantan Hospital, Capital Medical University, Beijing, China; ^2^ School of Pharmaceutical Sciences, Peking-Tsinghua Center for Life Sciences, Key Laboratory of Bioorganic Phosphorus Chemistry & Chemical Biology (Ministry of Education), Tsinghua University, Beijing, China; ^3^ Yufan Biotechnology (Beijing) Co., LTD, Beijing, China; ^4^ Advanced Innovation Center for Human Brain Protection, Beijing Tiantan Hospital, Capital Medical University, Beijing, China; ^5^ China National Clinical Research Center for Neurological Diseases (NCRC-ND), Beijing, China

**Keywords:** GD2, CARNK, anti-tumor efficacy, diffuse intrinsic pontine gliomas, pediatric

## Abstract

**Background:**

Diffuse intrinsic pontine gliomas (DIPGs) are rare and fatal pediatric brainstem gliomas with no cure. Chimeric antigen receptor (CAR)-engineered natural killer (NK) cells have been proven effective in treating glioblastoma (GBM) in preclinical studies. However, there are no relevant studies on the CAR-NK treatment for DIPG. Our study is the first to evaluate the anti-tumor activity and safety of GD2-CAR NK-92 cells treatment for DIPG.

**Methods:**

Five patient-derived DIPG cells and primary pontine neural progenitor cell (PPC) were used to access disialoganglioside GD2 expression. Cell killing activity of GD2-CAR NK-92 cells was analyzed by *in vitro* cytotoxicity assays. Two DIPG patient-derived xenograft models were established to detect the anti-tumor efficacy of GD2-CAR NK-92 cells *in vivo*.

**Results:**

Among the five patient-derived DIPG cells, four had high GD2 expression, and one had low GD2 expression. In *in vitro* assays, GD2-CAR NK-92 cells could effectively kill DIPG cells with high GD2 expression while having limited activity against DIPG cells with low GD2 expression. In *in vivo* assays, GD2-CAR NK-92 cells could inhibit tumor growth in TT150630 DIPG patient-derived xenograft mice (high GD2 expression) and prolong the overall survival of the mice. However, GD2-CAR NK-92 showed limited anti-tumor activity for TT190326DIPG patient-derived xenograft mice (low GD2 expression).

**Conclusion:**

Our study demonstrates the potential and safety of GD2-CAR NK-92 cells for adoptive immunotherapy of DIPG. The safety and anti-tumor effect of this therapy need to be further demonstrated in future clinical trials.

## 1 Introduction

Diffuse intrinsic pontine gliomas (DIPGs) are devastating pediatric brainstem tumors with no effective treatment (1). The median survival time of these patients is 10 months, and over 90% of patients with DIPG die within two years (2). Radiotherapy is the standard treatment protocol, but it only temporarily relieves symptoms and does not prolong overall survival (3). Given the poor clinical prognosis of DIPG, there is an urgent need to develop new treatments. In previous studies, disialoganglioside GD2 was demonstrated overexpression in DIPG cells, and GD2-CAR T cell therapy was proven effective against DIPG both *in vitro* and *in vivo* (4). However, neurologic toxicity and cytokine release syndrome (CRS) limit the widespread use of CAR T-cell therapy in brain tumors (5). CAR-NK cell therapy may be a better alternative to CAR-T because CAR-NK cells have better safety and higher feasibility for ‘off-the-shelf’ manufacturing than CAR-T cells (6).

In this article, we first analyzed the frequency of GD2 expression in five patient-derived DIPG cells and primary pontine neural progenitor cells (PPCs). The results showed that TT150630, TT150714, TT150728, and DIPG17 had high GD2 expression, TT190326 had low GD2 expression, and PPC had almost no GD2 expression. Then, we evaluated the anti-tumor activity of GD2-CAR NK-92 cells against these DIPG cells and PPC at different Effector/Target (E/T) ratios (1:1; 1:5, and 1:10). Furthermore, CD107a expression and IFN-γ production were evaluated. The results showed that GD2-CAR NK-92 cells could effectively kill the tumor cells for DIPG cells with high GD2 expression while exhibiting limited anti-tumor activity for DIPG cell with low GD2 expression. GD2-CAR NK-92 cells showed no cytotoxicity against PPC. Subsequently, the anti-tumor activity of GD2-CAR NK-92 cells against two orthotopic DIPG xenografts (TT150630 and TT190326) was evaluated. For TT150630 DIPG patient-derived xenograft mice, GD2-CAR NK-92 cells could inhibit tumor growth after the second-round treatment and prolong overall survival. For TT190326 DIPG patient-derived xenograft mice, the anti-tumor activity of GD2-CAR NK-92 cells was not significant.

## 2 Results

### 2.1 Construction of a GD2-specific CAR and generation of NK-92 cells expressing GD2-CAR

**Figure 1:**
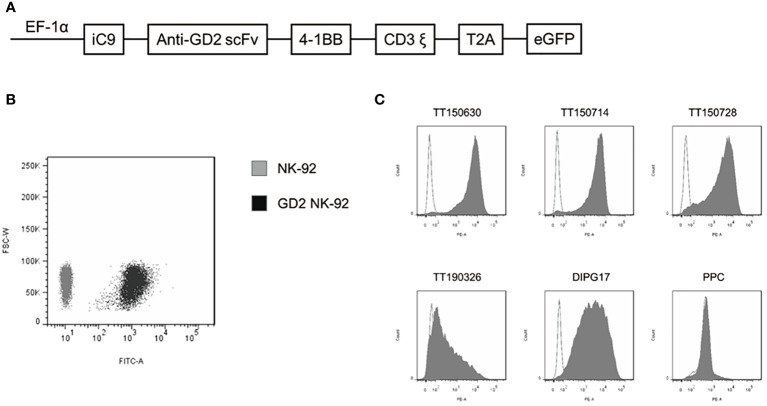
Construction of a GD2-specific CAR and expression of GD2 in DIPG cells. **(A)** Schematic representation of the GD2-CAR lentiviral construct. **(B)** GD2-CAR NK-92 cells were determined by flow cytometry with GFP (black area). NK-92 cells served as a control (gray area). **(C)** Expression of GD2 on the surface of five established DIPG cells and PPC was determined by flow cytometry with PE anti-human GD2-specific antibody.

The structure of GD2 CAR (scFv14G2a) incorporated with the 4-1BB and CD3ζ signaling domains, which was ligated into a lentiviral vector designated as pCDH-EF1-MCS-T2A-EGFP to generate a pCDH-EF1-iCAS9-GD2 CAR-4-1BB-CD3ζ-T2A-EGFP construct (**Figure 1A**). Then, we used the plasmid pMD2.G, psPAX2, and pCDH-CAR to produce lentivirus in Lenti-293T cells (Takala). Viral infection was performed in 12-well plates using 5×105 NK-92 cells in 1 mL of lentiviral supernatant containing 8 µg/mL polybrene (Sigma-Aldrich). Cells were centrifuged at 1000×g at 32°C for 50 min and then were incubated at 37°C for 6 h. EGFP-positive cells were sorted as GD2-CAR NK-92 cells by BD FACS Aria II about 72 h post transduction (**Figure 1B**). The transduction efficiency of NK-92 was approximately 4% (**Supplementary Figure 1A**). The lentiviral transduction is stable with only a minimal decrease in GD2-CAR expression observed by the MFI analysis of EGFP expression *via* FCM after one month. However, this was still about 100% of EGFP expression compared to that in untransduced NK-92 cells (**Supplementary Figure 1B**).

### 2.2 Expression of GD2 in primary DIPG cells and anti-tumor activity of GD2-CAR NK-92 cells against DIPG cells

**Figure 2:**
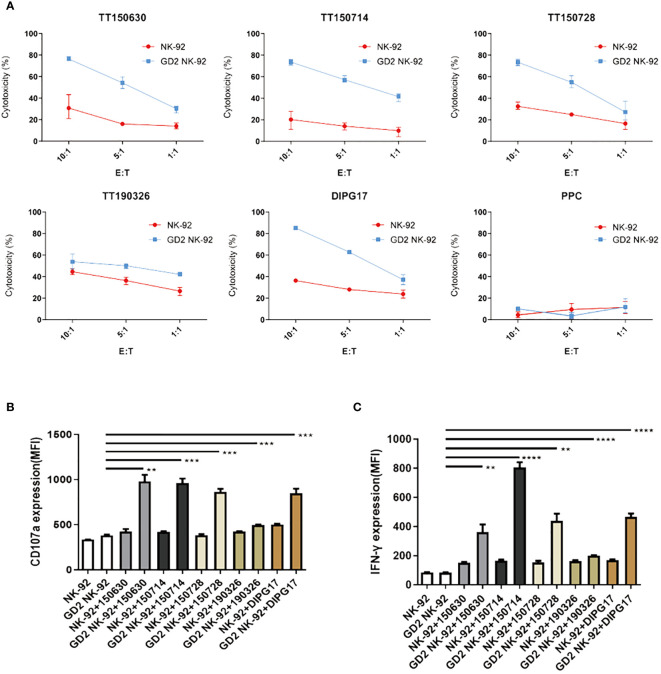
Specific cytotoxicity of GD2-CAR NK-92 cells against DIPG cells. **(A)** Cytotoxic activity of NK-92 or GD2-CAR NK-92 cells against five DIPG cells at three Effector/Target (E/T) ratios (1:1, 5:1, and 10:1) for 4 (h) **(B, C)** ELISA analysis of CD107a or IFN-γ expression by NK-92 and GD2-CAR NK-92 cells when co-cultured with five DIPG cells. Representative data from three independent experiments are shown. Unpaired t-tests were performed. P < 0.01 (∗∗), P < 0.001 (∗∗∗), and P < 0.0001 (∗∗∗∗).

Expression of GD2 on the surface of five DIPG cells (TT150630, TT150714, TT150728, TT190326, and DIPG17) and PPC were determined by flow cytometry with PE anti-human GD2-specific antibody. TT150630, TT150714, TT150728, and DIPG17 had a high GD2 expression, while TT190326 had a low GD2 expression, and PPC had almost no GD2 expression (**Figure 1C**). To access the anti-tumor activity of GD2-CAR NK-92 cells against DIPG cells *in vitro*, 5×10^4^ target cells including DIPGs and PPC were stained with CFSE, and then mixed with NK cells at different E:T ratios (1:1, 5:1, and 10:1) for 4 h at 37°C in a 5% CO2 atmosphere. Target cells alone served as spontaneous controls. After incubation, cells were washed with PBS and transferred to the absolute count microsphere test tube. Next, cells were added with viability dye Hoechst 33258, and the results were further analyzed using BD FACS Aria II. NK cytotoxicity (%) was calculated as ((TC - T)/TC)×100. GD2-CAR NK-92 cells could efficiently kill high-GD2 expression DIPG cells, showing a relative resistance to untargeted NK-92. In contrast, GD2-CAR NK-92 cells showed only marginal activity against low-GD2 expression DIPG cells (TT190326). GD2-CAR NK-92 cells and NK-92 cells showed no anti-tumor activity against PPC (**Figure 2A**). Likewise, GD2-CAR NK-92 cells co-cultured with DIPG cells produced more IFN-γ and CD107a than mock-transduced NK-92 cells (**Figures 2B, C**). These results indicate that GD2-CAR NK-92 cells can significantly enhance NK cell cytotoxicity and IFN-γ and CD107a production against GD2-high expression DIPG cells compared to unmodified NK-92 cell controls.

### 2.3 Anti-tumor activity of GD2-CAR NK-92 cells against orthotopic DIPG xenografts

**Figure 3:**
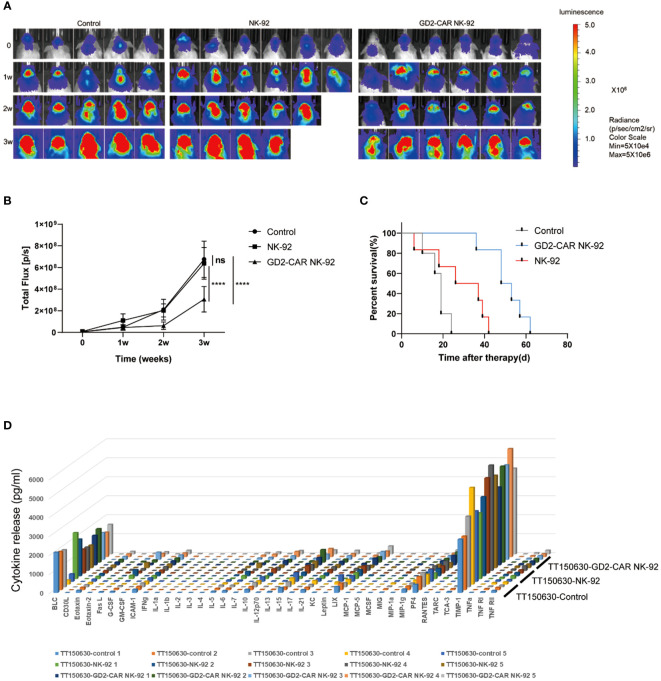
The potent anti-tumor efficacy of GD2-CAR NK-92 cells in NCG mice bearing TT150630 DIPG cells (high GD2 expression). **(A, B)** Luciferase-containing engineered TT150630 DIPG cells were implanted into the brainstem of NCG mice. One week later, mice with established orthotopic TT150630 DIPGs were randomly assigned to control, NK-92 cells treated, and GD2-CAR NK-92 cells-treated groups, respectively. PBS, 3×10^6^ NK-92 cells, and 3×10^6^ GD2-CAR NK-92 cells were injected into the lateral ventricle of NCG mice weekly for two weeks. Normalized luminescence was analyzed by Living Image software Version 3.0 (Caliper Life Sciences). **(C)** Kaplan–Meier survival curve plotting. Log-rank test was performed to compare survival between the groups. **(D)** Release assay of various cytokines in the plasma from NCG mice bearing TT150630 DIPG cells. Mice were treated with PBS, NK-92 cells, and GD2-CAR NK-92 cells, respectively. Three days later, mice were sacrificed to collect plasma and levels of indicated cytokines were measured by a cytokine release array assay. P < 0.0001 (∗∗∗∗). NS, No Significance.

**Figure 4:**
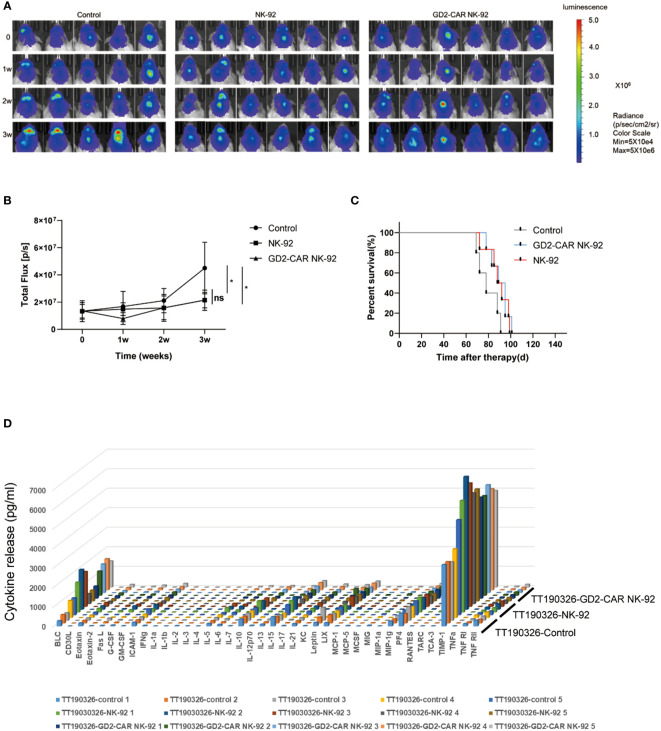
The anti-tumor efficacy of GD2-CAR NK-92 cells in NCG mice bearing TT190326 DIPG cells (low GD2 expression). **(A, B)** Luciferase-containing engineered TT190326 DIPG cells were implanted into the brainstem of NCG mice. Two weeks later, mice with established orthotopic TT190326 DIPGs were randomly assigned to control, NK-92 cells treated and GD2-CAR NK-92 cells-treated groups, respectively. PBS, 3×10^6^ NK-92 cells, and 3×10^6^ GD2-CAR NK-92 cells were injected into the lateral ventricle of NCG mice weekly for two weeks. Normalized luminescence was analyzed by Living Image software Version 3.0 (Caliper Life Sciences). **(C)** Kaplan–Meier survival curve plotting. Log-rank test was performed to compare survival between the groups. **(D)** Release assay of various cytokines in the plasma from NCG mice bearing TT190326 DIPG cells. Mice were treated with PBS, NK-92 cells, and GD2-CAR NK-92 cells, respectively. Three days later, mice were sacrificed to collect plasma and levels of indicated cytokines were measured by a cytokine release array assay. P < 0.05 (∗). NS, No Significance.

Two luciferase-modified DIPG cells, TT150630 (high-GD2 expression) and TT190326 (low-GD2 expression) were used to establish orthotopic DIPG mouse models. DIPG cells were injected into the brainstem of NCG mice, and the bioluminescence images were acquired after 10 days. The mice were randomly divided into control, NK-92 cells-treated, and GD2-CAR NK-92 cells-treated groups. There was no statistical difference in tumor volume among the three groups. Then, 10 μl PBS, 3×10^6^ NK-92 cells, and 3×10^6^ GD2-CAR NK-92 cells were injected into the lateral ventricle of the mice weekly for two consecutive weeks, respectively. The bioluminescence images were acquired weekly to measure the tumor volume. For TT150630 orthotopic DIPG mice, significant inhibition of tumor growth was observed in the GD2-CAR NK-92 cells-treated group compared with the control group (P<0.0001) and NK-92 cells-treated group (P<0.0001) (**Figures 3A, B**). The overall survival was significantly longer in the GD2-CAR NK-92 cells-treated group than that in the control group (P = 0.0008) and NK-92 cells-treated group (P = 0.0049) (**Figure 3C**). For TT190326 orthotopic DIPG mice, inhibition of tumor growth was observed in the GD2-CAR NK-92 cells-treated group compared with the control group (P = 0.0404) and NK-92 cells-treated group (P = 0.0310). (**Figures 4A, B**). However, there is no significant statistical difference in overall survival between GD2-CAR NK-92 cells-treated group and the control group or NK-92 cells-treated group (**Figure 4C**).

### 2.4 Safety profiling in NCG mice

To evaluate the safety profile of GD2-CAR NK-92 cells *in vivo*, NCG mice bearing TT190326 and TT150630 DIPG cells were treated with PBS, NK-92 cells, and GD2-CAR NK-92 cells, respectively. Three days later, these mice were sacrificed to collect plasma and levels of indicated cytokines were measured by a cytokine release array assay. The results showed that the levels of the 40 cytokines, including IL6, IFN-γ, and TNF-γ, had no significant difference among the four treatment groups (**Figures 3D**, **4D**), suggesting that cytokine release syndrome (CRS) do not occur during the treatment. Brain, heart, liver, spleen, lung, and kidney were collected from the DIPG xenograft mice after a 2-day treatment. No acute injury was observed in the HE sections of these organs (**Supplementary Figure 2**).

### 2.5 Assessment of the persistence of CAR NK-92 cells

In clinical trials, it is necessary to irradiate CAR NK-92 cells with 10Gy before infusion (7). In order to assess the persistence of irradiated CAR NK-92 cells for potential clinical applications in DIPG therapy, we conducted viability tests. Under a microscope, the irradiated and non-irradiated states of CAR NK-92 cells in culture medium (CM) or cerebrospinal fluid (CSF) can be observed (**Supplementary Figure 3A**). Our findings indicate that irradiated CAR NK-92 cells exhibit a decrease in viability of approximately 20%, 50%, and 85% on Day1, Day2, and Day3 post-irradiation, respectively, and almost no activity on Day4 post-irradiation (**Supplementary Figure 3B**). The cytotoxicity activity against target cells demonstrated a decline of about 40% and 80% on Day1, Day2 post-irradiation at E/T ratio of 1:10, respectively (**Supplementary Figure 3D**). Due to the limited amount of cerebrospinal fluid that can be extracted from mice, which is only 40μL (8), evaluating CAR NK-92 cells persistence *in vivo* is challenging. To address this, we used artificial cerebrospinal fluid to simulate the *in vivo* environment and assess the persistence of CAR NK-92 cells in artificial cerebrospinal fluid. Our results indicate that non-irradiated CAR NK-92 cells showed almost unchanged viability on Day1, and a subsequent decline by approximately 35%, 80%, and 90% on Day2, Day3 and Day4, respectively (**Supplementary Figure 3C**). The cytotoxicity activity against target cells displayed a decrease of approximately 16%, 33% and 85% on Day1, Day2 and Day3 at E/T ratio of 1:10, respectively. On the other hand, irradiated CAR NK-92 cells showed a decrease in viability by approximately 20% and 80% on Day1 and Day2 post-irradiation, respectively, with almost undetectable viability on Day3 post-irradiation. The cytotoxicity activity against target cells exhibited a decline of approximately 80% on Day1 post-irradiation, and almost no activity on Day2 post-irradiation (**Supplementary Figure 3D**).

## 3 Discussion

Diffuse intrinsic pontine gliomas (DIPGs) comprise 80% of pediatric brainstem gliomas, and the median survival is less than one year (9, 10). The understanding of DIPG has made many breakthroughs in molecular biology. One of the most important findings is that 70%–84% of DIPG possess mutations in histone H3 (11). However, radiotherapy remains the standard of care for DIPG, and no other treatment protocol has been proven effective for DIPG (12). Disialoganglioside GD2 is a well-suited target for cancer therapy because of its high density on tumor cells and restricted expression on normal tissues (13). In addition to neuroblastoma, GD2 is overexpressed in many solid tumors, including retinoblastoma, Ewing’s family of tumors, and DIPG (4, 13). In previous studies, GD2 is overexpressed in DIPG cells. GD2-CAR T cell therapy is effective in DIPG both *in vitro* and *in vivo* (4), and interestingly, it has also shown potential in patients with DIPG in subsequent clinical trials (14).

CAR-NK cell therapy has recently been found promising in attacking malignant tumors (6). Preclinical studies of CAR-NK cell therapy demonstrated its enormous potential in hematological tumors such as B-lineage malignant cells and multiple myeloma (MM) (15, 16). CAR NK-92 cells also show an effect in attacking solid tumors, including neuroblastoma, osteosarcoma, hepatocellular carcinoma (HCC), pancreatic cancer, and breast cancer (17, 18). Thirteen studies on CAR-NK cells treating solid tumors have been registered on http://www.clinicaltrials.gov; however, these clinical trials have not yet had definitive results. There are two studies reporting CAR-NK treatment of glioblastoma (GBM). Zhang et al. (19) reported the targeted therapy of ErbB2/HER2-specific NK cells for GBM. The intratumoral injection of 2×10^6^ ErbB2-specific NK-92/5.28.z cells was performed once a week for 11 weeks, and significant inhibition of tumor growth was observed. Han et al. (20) used the EGFR-CAR NK-92 cells against GBM and injected 2×10^6^ CAR-NK cells locally on day 10, day 40, and day 70 after the animal model construction. Montagner and Penna et al. studied CARNK-92 in the treatment of prostate cancer, especially the application of irradiated CARNK-92, which is closer to clinical practice (21).

Our study is the first to evaluate the safety and anti-tumor activity of GD2-CAR NK-92 cells against DIPGs. GD2-CAR NK-92 cells showed specific activity against DIPG cells with significant anti-tumor activity against high-GD2 expression DIPG cells but limited activity against low-GD2 expression DIPG cells. NK can only inhibit the growth of mouse tumors *in vivo* and cannot completely relieve brain tumors like CAR-T (4). However, the strong curative effect of CAR-T is also accompanied by some adverse reactions, such as cytokine storm and cerebral edema. For DIPG that grows in the brainstem, cerebral edema may cause fatal brain herniation. Although the efficacy of CAR-NK cells is not as good as that of CAR-T, we have not found adverse reactions such as cytokine storm and cerebral edema. From the perspective of safety, the safety of CAR-NK in treating DIPG may be better than that of CAR-T. It is important to note that the use of NCG mice, a severe immunodeficiency mouse, may not be the most appropriate animal model for studying cytokine release syndrome (CRS). Instead, animal models such as SCID/Beige or humanized models may offer better insights into CRS pathogenesis. Therefore, we recommend using more appropriate animal models to study CRS in future studies. In addition, Mount et al. (4) found graft-versus-host reaction in the DIPG mouse model treated with CAR-T. In our experiment, the mice did not show extensive hair loss, which may suggest a graft-versus-host reaction. Considering the fragility of the brainstem, we reinfused CAR-NK cells into the lateral ventricle instead of the brainstem in situ. The efficacy of CAR-NK may be limited because the cells reinfused from the ventricle may not 100% infiltrate into the tumor.

The previous clinical trial irradiated the cells with a dose of 10 Gy before infusing CAR NK-92 cells (7). We also irradiated CAR NK-92 cells with a dose of 10 Gy and found that after 2 days of irradiation, the cells almost lost their ability to kill target cells. The impact of irradiation on CAR NK-92 cells needs to be considered in future clinical trials in DIPG, and shortening the time interval between infusions may help improve the therapeutic efficacy.

## 4 Conclusion

The safety and anti-tumor activity of GD2-CAR NK-92 cells against DIPG cells have been determined in this study. CAR-NK cell therapy has shown potential in treating DIPG, which needs to be further verified in future clinical trials.

## 5 Materials and methods

### 5.1 Construction of GD2-CAR NK-92 cells

The structure of GD2 CAR (scFv14G2a) incorporated with the 4-1BB and CD3ζ signaling domains, which was ligated into a lentiviral vector designated as pCDH-EF1-MCS-T2A-EGFP (Xi’an Yufan Biotechnologies, Shaanxi, China) to generate a pCDH-EF1-iCAS9-GD2 CAR-4-1BB-CD3ζ-T2A-EGFP construct. The lentivirus production and transduction of NK-92 cells were modified from a previously published protocol [https://www.nature.com/articles/leu2013279#Sec2]. Briefly, the plasmid pMD2.G, psPAX2, and pCDH-CAR produced lentivirus in Lenti-293T cells (Takala). Viral infection was performed in 12-well plates using 5×105 NK-92 cells in 1 mL of lentiviral supernatant containing (MOI: 10) 8 µg/mL polybrene (Sigma-Aldrich). Cells were centrifuged at 1000 × g at 32°C for 50 min and then were incubated at 37°C for 6 h. EGFP-positive cells were sorted as GD2-CAR NK-92 cells by BD FACS Aria II about 72 h after transfection.

### 5.2 Cell establishment and culture conditions

The patient-derived DIPG cells (TT150630, TT150714, TT150728, and TT190326) and DIPG17 cells were used in this study. The cell culture method was reported previously (19). Human NK cell lines NK-92 were cultured with a special culture medium for NK-92 cell lines (TCH-G293), including MEM α, 0.2 mM inositol, 0.1 mM β-mercaptoethanol, 0.02 mM folic acid, 12.5% HS, 12.5% FBS, 1% P/S, and 150 IU/mL recombinant human (rh) IL-2 (Hycyte). All the cells were cultured at 37°C in a 5% CO2 atmosphere. Cells were analyzed by flow cytometry for GD2 expression using PE anti-human Ganglioside GD2 [14G2a] from biolegend.

### 5.3 Flow cytometry-based cytotoxicity

A total of 5×10^4^ target cells, including DIPGs and PPC, were stained with CFSE, then mixed with NK cells at different E:T ratios (1:1, 5:1, and 10:1) for 4 h at 37°C in a 5% CO2 atmosphere. Target cells alone served as spontaneous controls. After incubation, cells were washed with PBS and transferred to the absolute count microsphere test tube (No. Z6410004). Next, cells were added with viability dye Hoechst 33258 (ab228550), and the results were further analyzed using BD FACS Aria II. NK cytotoxicity (%) was calculated as ((TC - T)/TC) × 100. In control tubes, TC means the absolute number of live CFSE+ target cells (CFSE+ Hoechst 33258-). T is the absolute number of live CFSE+ target cells in test tubes.

### 5.4 Luciferase-based cytotoxicity

To investigate the cytotoxicity of GD2 CARNK cells against luciferase-engineered TT150630 cells, co-incubation experiments were conducted. Specifically, 10^4^ TT150630 cells in 50μL were co-cultured with 50μL of GD2 CARNK cells at different effector-to-target (E:T) ratios of 1:1, 5:1, and 10:1 for 4 hours at 37°C in a 5% CO2 atmosphere, using a 96-well plate. Control groups were performed using only target cells. After incubation, 100μL of 300μg/ml D-Luciferin (LUCK-1G) was added to each well, and triplicate wells were used for each experiment. Fluorescence was measured with a microplate reader, and the cytotoxicity (%) was calculated using the formula ((FC - FE)/FC)×100, where FC represents the fluorescence value of the control group and FE represents the fluorescence value of the experimental group.

### 5.5 Assessment of CAR NK-92 cells activity with or without irradiation

2×10^4^ CAR NK-92 cells with irradiation (10Gy) and without irradiation were counted on Day 0, Day 1, Day 2, Day 3, and Day 4 in culture medium (TCH-G293) or artificial cerebrospinal fluid (SSL6630), respectively. Cell viability was assessed using the CellTiter-Glo luminescent cell viability assay (G7572). The cell viability (%) was calculated as V0/VD, where V0 was the cell viability before irradiation, and VD was the cell viability in Day 0, Day 1, Day 2, Day 3 and Day 4.

### 5.6 Detection of CD107a expression and IFN-γ production

To detect degranulation and IFN-γ production of NK cells, NK cells were cultured with target cells at a 1:1 ratio for 5 h in the presence of GolgiStop (BD Biosciences) and PE-labeled anti-CD107a antibody (Biolegend). After incubation, cells were washed and stained with APC labeled anti-CD56 and FITC labeled anti-CD3 and then were fixed/permeabilized with Cytofix/Cytoperm Buffer (BD Biosciences). BV421 labeled anti-IFN-γ(Biolegend) was used to stain cells for the detection of intracellular IFN-γ.

### 5.7 Establishment of DIPG xenograft mice models and lateral ventricle injection of GD2-CAR NK-92 cells

Six-week female NCG mice were used to establish DIPG xenograft mouse models. TT150630 and TT190326 tumor cells were injected into the brainstem of the mice. The method for establishing the animal model was reported previously (22). The tumor burden was measured by bioluminescence imaging 2 weeks after the implantation. The tumor-bearing mice were randomly assigned to the control, NK-92 cells, and GD2-CAR NK-92 cells treated groups. Then, 10 μl PBS, 3×10^6^ NK-92 cells, and 3×10^6^ GD2-CAR NK-92 cells were injected into the lateral ventricle of the mice every week for two consecutive weeks, respectively. Biofluorescence and body weight were measured weekly for all mice. After injecting 10 μl PBS, 3×10^6^ NK-92 cells, and 3×10^6^ GD2-CAR NK-92 cells into the lateral ventricle of the mice for three days, the release of various cytokines in the plasma from DIPG xenograft mice was evaluated using Mouse Inflammation Array Q1 (QAM-INF-1).

### 5.8 Hematoxylin and eosin staining

Brain, heart, liver, spleen, lung, and kidney were collected from the DIPG xenograft mice after three days of treatment. The methods for H&E (Hematoxylin-Eosin) staining were performed as previously reported (23).

### 5.9 Statistical analysis

Graphpad prism 8 was utilized for statistical analysis. Statistical significance was calculated by an unpaired two-tailed t-test between groups. P < 0.05 was considered statistically significant: P < 0.05 (∗), P < 0.01 (∗∗), P < 0.001 (∗∗∗), and P < 0.0001 (∗∗∗∗).

## Data availability statement

The original contributions presented in the study are included in the article/**Supplementary Material**. Further inquiries can be directed to the corresponding authors.

## Ethics statement

The studies involving animals were reviewed and approved by the Animal Welfare Ethics Committee of Beijing Neurosurgical Institute.

## Author contributions

Writing - Original Draft: PZ. Methodology: YL. Formal analysis: CH, TW. Data Curation: XZ, HL, ZW, JZ. Resources Writing - Review & Editing: XL, LZ. All authors contributed to the article and approved the submitted version.
