# Supplementary material for: Anti-tumor efficacy of anti-GD2 CAR NK-92 cells in diffuse intrinsic pontine gliomas
Source: Front Immunol. 2023 May 12;14:1145706. doi: 10.3389/fimmu.2023.1145706 (PMC10213244; doi:10.3389/fimmu.2023.1145706)
Supplement: Supplementary file 1 [file DataSheet_1.doc]

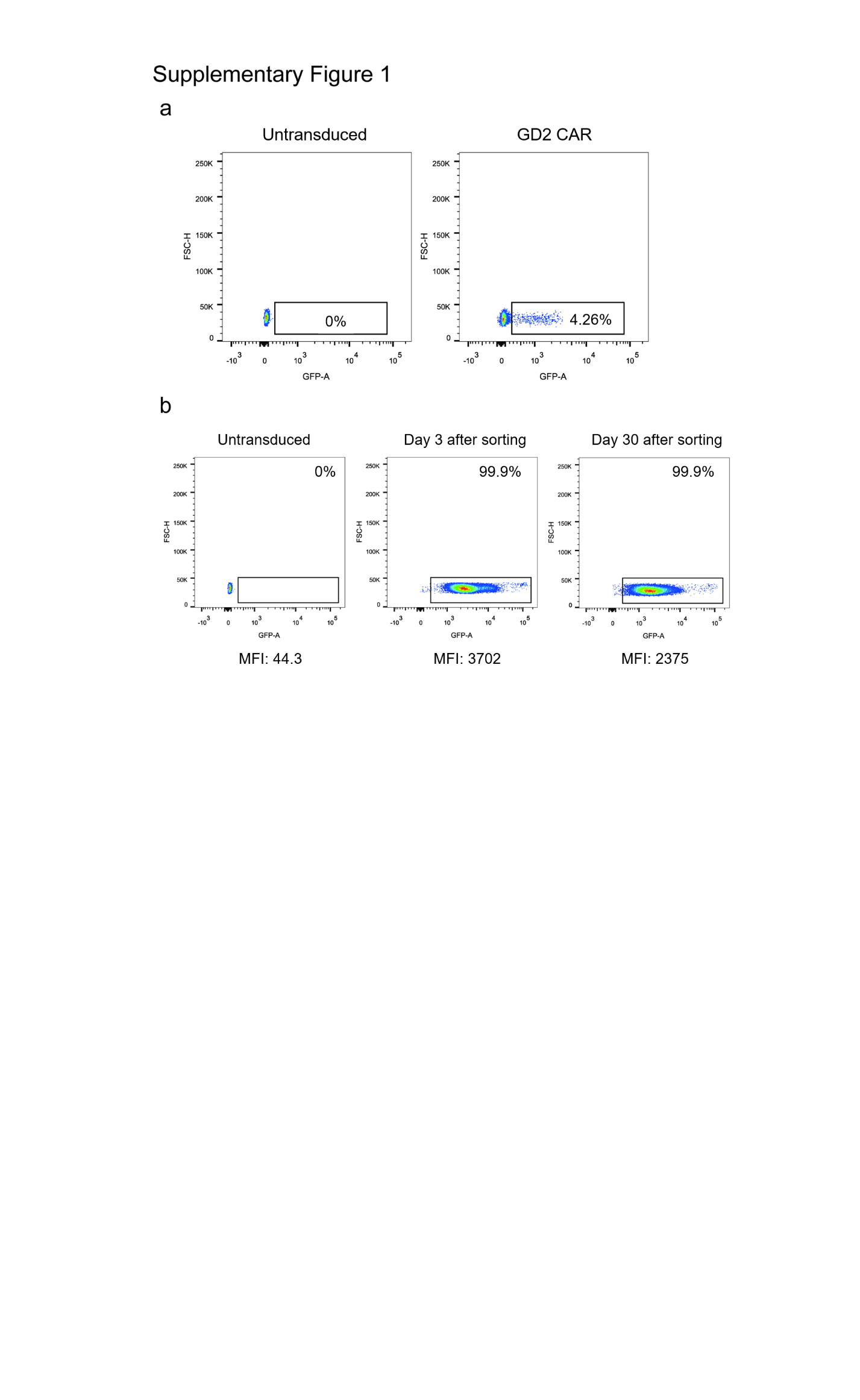


(a) The transduction efficiency (CAR% expression) of NK-92. (b) GD2-CAR expression after 3 days, 30 days post transduction.


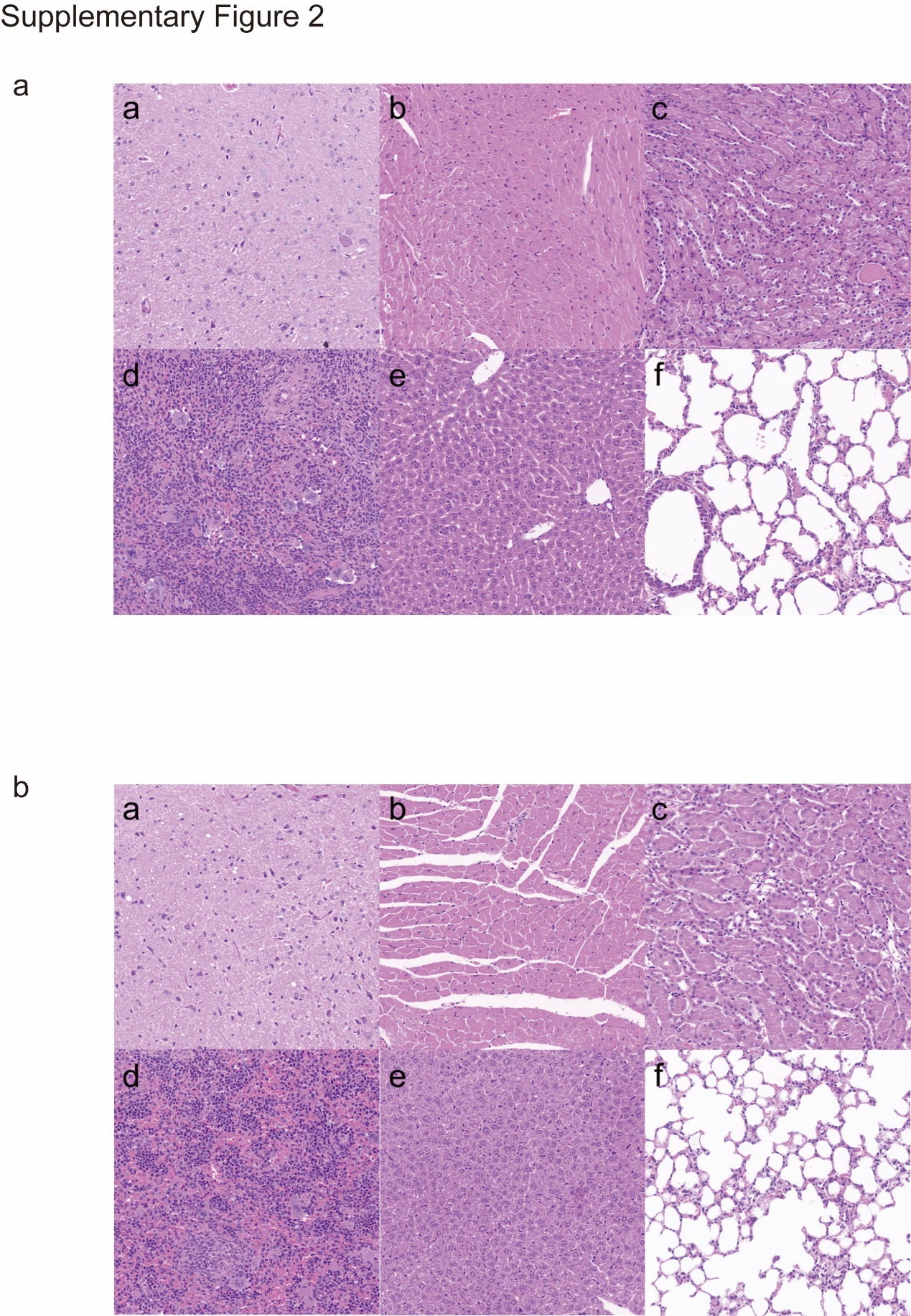


(a) Brain (a), heart (b), kidney (c), spleen (d), liver (e), lung (f) and were collected from TT150630 Orthotopic DIPG mice and from TT190326 Orthotopic DIPG mice (b).


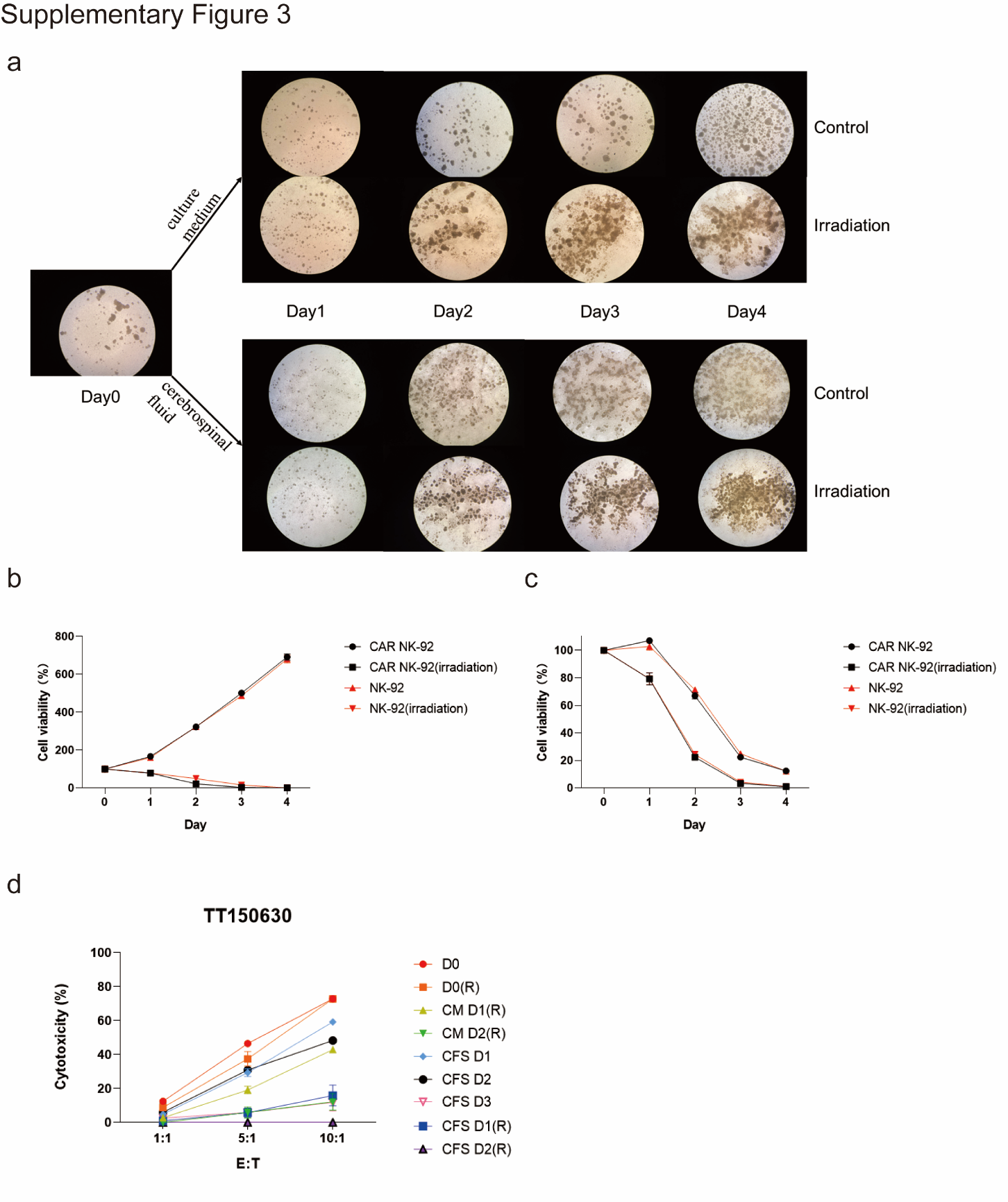
(a) The irradiated or non-irradiated state of CAR NK-92 cells in culture medium (CM) or cerebrospinal fluid (CSF) under a microscope. (b) cell viability of CAR NK-92 cells or NK-92 cells with irradiation or without irradiation in CM. (c) cell viability of CAR NK-92 cells or NK-92 cells with irradiation or without irradiation in CSF. (d) Cytotoxicity of CAR NK-92 cells with or without irradiation against TT150630 cells in CM or CFS.
